# Systematic review of the psychometric properties of instruments to measure sexual desire

**DOI:** 10.1186/s12874-018-0570-2

**Published:** 2018-10-19

**Authors:** Denisse Cartagena-Ramos, Miguel Fuentealba-Torres, Flávio Rebustini, Ana Carolina Andrade Biaggi Leite, Willyane de Andrade Alvarenga, Ricardo Alexandre Arcêncio, Rosana Aparecida Spadoti Dantas, Lucila Castanheira Nascimento

**Affiliations:** 1 Escola de Enfermagem de Ribeirão Preto – EERP-USP, Universidade de São Paulo, Av. Bandeirantes, 3900, Ribeirão Preto, São Paulo, 14040-902 Brazil; 2 Escola de Artes, Ciências e Humanidades, Universidade de São Paulo, São Paulo, SP Brazil

**Keywords:** Libido, Sexual desire, Psychometric, Validity, Reliability, Reproducibility of results, Cross-cultural comparison

## Abstract

**Background:**

Sexual desire is one of the domains of sexual function with multiple dimensions, which commonly affects men and women around the world. Classically, its assessment has been applied through self-report tools; however, an issue is related to the evidence level of these questionnaires and their validity. Therefore, a systematic review addressing the available questionnaires is really relevant, since it will be able to show their psychometric properties and evidence levels.

**Method:**

A systematic review was carried out in the PubMed, EMBASE, PsycINFO, Science Direct, and Web of Science databases. The search strategy was developed according to the following research question and combination of descriptors and keywords, including original studies with no limit of publication date and in Portuguese, English, and Spanish. Two reviewers carried out the selection of articles by abstracts and full texts as well as the analysis of the studies independently. The methodological quality of the instruments was evaluated by the COnsensus-based Standards for the selection of health status Measurement INstruments (COSMIN) checklist.

**Results:**

The search resulted in 1203 articles, of which 15 were included in the review. It identified 10 instruments originally developed in the English language. Unsatisfactory results on methodological quality were evidenced in cultural adaptation studies with no description of the steps of the processes and inadequacy of techniques and parameters of adequacy for models. The Principal Component Analysis with Varimax rotation predominated in the studies.

**Conclusions:**

The limitation of the techniques applied in the validation process of the reviewed instruments was evident. A limitation was observed in the number of adaptations conducted and contexts to which the instruments were applied, making it impossible to reach a better understanding of the functioning of instruments. In future studies, the use of robust techniques can ensure the quality of the psychometric properties and the accuracy and stability of instruments. A detailed description of procedures and results in validation studies may facilitate the selection and use of instruments in the academic and/or clinical settings.

**Systematic review registration:**

PROSPERO CRD42018085706.

**Electronic supplementary material:**

The online version of this article (10.1186/s12874-018-0570-2) contains supplementary material, which is available to authorized users.

## Background

Sexual desire has been defined as the force to stimulate or inhibit sexual behavior [1], as well as interest in sexual activity [2], and the literature also recognises it as one of the sexual response cycle phases [3] and one of the domains of sexual function [4]. Beyond, sexual desire may be understood by the biological, psychological and social components [1].

According to authors, there are three models of self-reports to evaluate sexual behavior evaluation by interview, questionnaires and behavioural records filled by the client or subject [5, 6]. The assessment by self-report is more widely and commonly applied to measure the sexual desire and functioning.

Systematic reviews on psychometric properties for sexual (dys)function have been carried out to identify available measurement instruments [7, 8]; however, those addressing sexual desire are still limited.

Regarding sexual desire and functioning, different questionnaires applied to diagnose hypoactive sexual desire disorders as well estimating their real prevalence and associated factors and magnitude have been observed [9, 10].

Therefore, the use of instruments to evaluate sexual desire, among other dimensions of sexual life, can be helpful in the planning of multidisciplinary interventions aimed at helping individuals that present with this disorder. Thus, assessing the quality of instruments that measure sexual desire is an essential step in revealing the positive and negative aspects of measurements and providing evidence-based guidance for the selection of validated instruments for the academic and/or clinical contexts.

The COSMIN Checklist is a tool that is increasingly used in systematic analyses to evaluate the measurement properties of instruments [11, 12]. This tool was chosen in the present systematic review with the objective of evaluating the methodological quality of the psychometric properties and the level of evidence of the selected instruments that measure sexual desire.

## Method

A systematic review of measurement instruments was conducted according to the ten steps of the COSMIN protocol [13]: 1) formulation of the research question; 2) literature search; 3) selection criteria; 4) selection of articles by abstracts and full texts; 5) evaluation of the methodological quality of included studies; 6) extraction of the data; 7) content comparison; 8) data synthesis and evaluation of instruments quality; 9) general conclusion of the systematic review; and 10) preparation of the report on the psychometric properties of the evaluated instruments.

### Search strategy

A systematic review was carried out in the PubMed, EMBASE, PsycINFO, Science Direct, and Web of Science databases. The following search strategy was performed in PubMed using MESH in combination with the following keywords: (libido) AND (psychometrics) AND (cross cultural-comparison) OR (cross-cultural AND comparison) OR (cross AND cultural AND comparison) AND (sexual desire) OR (sexual AND desire) OR (sexual AND interest) OR (sexual interest). This search strategy was adapted to the other databases. [See Additional file 1] for the complete search strategy. All citations were imported into the bibliographic database of EndNote Basic.

### Selection criteria

The inclusion criteria were established as: original studies, published in Portuguese, English, and Spanish with human beings, and presenting the process of evaluating cultural validations and adaptations of sexual desire instruments, regardless of sample sex or gender. There was no limitation on the initial date of publication, and studies published until November 2017 were considered. In addition, it was determined that articles presenting the dimension of sexual desire or the condition of its decrease (hypoactive sexual desire disorder) would also be included. Articles that aimed to measure dysfunctions in other dimensions of the sexual response in men and/or women and samples with paediatric population were excluded.

### Selection of articles by abstracts and full texts

The selection of articles by abstracts and full texts were performed independently by two reviewers (DC and MF), according to the selection criteria. All studies retrieved were imported into the bibliographic database of EndNote Basic. Then, the references were exported to Microsoft Excel, version 2016. In case of disagreement in the selection of the studies, two others reviewers (LC and RA) were consulted.

### Data extraction and synthesis

The data extraction of potentially eligible literature were performed independently by two reviewers (DC and MF), and they extracted the following data: author, year of publication, country, title of the study, source, inclusion criteria, exclusion criteria, items, average fill time, population and sample size (n), and types of psychometric properties tested. [See Additional file 2].

**Fig. 1:**
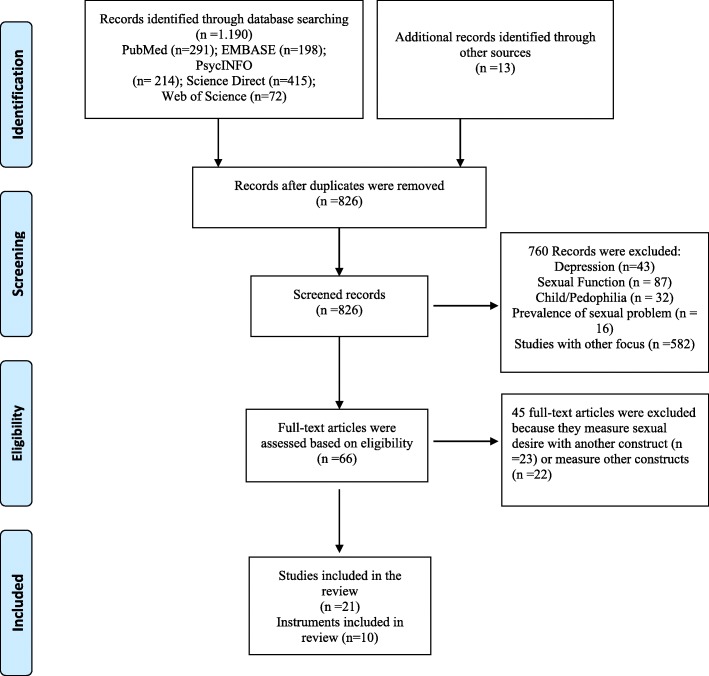
Flowchart of the studies included in the systematic review

The reviewers identified 1190 articles. Another 13 articles were captured through a manual search of references reported in the articles identified first, totalling 1203 articles; of these, 826 were duplicates and not included in the study. The titles and abstracts of 66 studies were analysed by two independent reviewers (DC and MF); in case of disagreement in the selection of the studies, two other reviewers (LC and RA) were consulted. In the end, 66 articles were considered adequate for inclusion in the study. The inter-observer agreement was measured by the Kappa test, with a score of 0.84. Subsequently, the 66 articles were analysed in their entirety and separately by two reviewers (DC and MF). A total of 45 articles were excluded based on the following reasons: they measured sexual desire together with other dimensions of sexual function (*n* = 23) or measured other constructs (*n* = 22). Therefore, 21 articles were included in the study, with a total of 10 instruments that measured sexual desire. The search and selection process is presented in Fig. 1 using the PRISMA flowchart [14].

### Evaluation of methodological quality

Two reviewers (DC and MF) independently applied the COSMIN Checklist [12] to evaluate the methodological quality of the psychometric properties reported in the included studies. Discordances between the two reviewers were resolved with the participation of a third reviewer who is an expert in psychometrics (FR). The COSMIN Checklist was developed through the international Delphi study [15] in order to facilitate the methodological evaluation of outcome measures for the proper choice of an instrument. This checklist includes nine evaluation parameters: internal consistency, reliability, measurement error, content validity, construct validity, hypothesis testing, cultural validity, criterion validity, and responsiveness.

The quality of psychometric properties was evaluated by a number of items, including design and preferred statistical methods requirements. A four-point rating scale (poor, fair, good, and excellent) was used for the evaluation depending on the information reported by the study authors. A total score was determined according to the lowest item ranking for each measurement property [12].

### Synthesis and levels of evidence

After the evaluation by the COSMIN Checklist, the results were combined by instrument to determine the level of evidence of the analysed studies according to the methodological quality criteria of the studies [16] and classified according to the criteria proposed by the Cochrane Back Review Group [17] as: strong (consistent positive results from multiple studies with good methodological quality or one study with excellent methodological quality), moderate (consistent positive results from multiple studies with fair methodological quality or one study with good methodological quality), limited (positive results from a study with fair methodological quality), conflicting (conflicting results from individual studies), or unknown (results from studies with poor methodological quality with an unknown level of evidence).

## Results

Out of a total of 1203 articles identified, 21 were included in the review, in which 10 instruments were identified. The search and selection processes are presented in the Fig. 1, using the PRISMA flowchart [14].

All the studies included in the systematic review were documented as supplemental references and were identified in the text with the prefix ‘s’, followed by the respective reference number. [Additional file 3].

### The characteristics of the included studies are presented bellow (Table 1)

The results of the COSMIN evaluation (Table 2), and evidence levels (Table 3) of instruments are presented.

**Table 1:** The characteristics of the included studies

Instrument	Country	Items	Average fill time	Population	Sample (n)
*Questionnaire Measure of Sexual Interest (QMSI)* [57]	Ireland	140	20 min	Women and Men	94
*Sexual Desire Conflict Scale for Women (SDCSW)* [18]	USA	33	Not reported	Women	54
*Sexual Desire Inventory (SDI-2)* [2]	Canada	14	Not reported	Women and Men	380
*Sexual Desire Inventory (SDI-2)* [25]	Germany	13	Not reported	Women and Men	156
*Sexual Desire Inventory (SDI-2)* [26]	Spain	14	Not reported	Women and Men	608
*Sexual of Fantasy Questionnaire (SFQ)* [62–64]	England	40	Not reported	Women and Men	90 [22–24]
*Sexual of Fantasy Questionnaire (SFQ)* [65, 66]	Spain	32	Not reported	Women and Men	Two samples *n* = 460 [25] and *n* = 510 [26]
*Menopausal Sexual Interest Questionnaire (MSIQ)* [19]	USA	10	Not reported	Women	332
*The Sexual Interest and Desire Inventory-Female (SIDI-F)* [20, 21, 67, 68]	USA	13	5 min	Women	448 [31]
*The Sexual Interest and Desire Inventory-Female (SIDI-F)* [28]	Iran	13	15 min	Women	Three samples: *n* = 90 [28], *n* = 248 [29] and *n* = 428 [30] 40
*Screener for Hypoactive Sexual Desire Disorder in Menopausal Women (SHSDD)* [22]	USA	4	Not reported	Women	959
*Cues for Sexual Desire Scale CSDS* [23]	USA	40	Not reported	Women	Two samples *n* = 874 and *n* = 138
*Cues for Sexual Desire Scale CSDS* [27]	Portugal	40	Not reported	Women	3.687
*The Sexual Arousal and Desire Inventory (SADI)* [69]	USA	54	Not reported	Women and Men	390
*Female Sexual Desire Questionnaire (FSDQ)* [24]	Australia	50	30 min	Women	741

**Table 2:** Results of the psychometric properties of the instruments included and rated by the COSMIN checklist

Instrument	COSMIN Assessment	Validity/Reliability
*Questionnaire Measure of Sexual Interest (QMSI)* [57]	Box A: Poor Box B: Poor Box C: NR Box D: Poor Box E: Poor Box F: NR Box G: NA Box H: NR Box I: NR	**Content Validity:** This is the standard pattern for the questionnaire; the scale positions within each pair were then randomized. As there are four bipolar adjectives and five levels of sexual behavior to be rated, the questionnaire comprises 140 items. These are set out in random order, in the form of a questionnaire. **Construct Validity:** PCA identified five components. Factor loadings between 0.83–0.98; *p* < 0.001. With variances ranging from 69.2 to 92.9% for all three groups. **Internal Consistency:** Kuder Richardson Coefficient between 0.83–0.95. **Test Retest:** 0.68 to 0.92 (*p* < 0,01 or *p* < 0.001).
*Sexual of Fantasy Questionnaire (SFQ)* [62–64]	Box A: Fair Box B: NR Box C: NR Box D: Poor Box E: Poor Box G: Poor Box H: NR Box I: NR	**Content Validity:** A questionnaire was developed consisting of 40 recognized fantasy themes drawn from scientific and erotic literature [2]. **Construct Validity:** PCA and varimax rotation identified four components. With a correlation of *r* = 0.04 to 0.32. Total variance explained by 45.47%. Factorial loads ≥0.30 and communalities that vary between 0.14–0.71. **Internal Consistency:** Cronbach’s alpha between 0.69–0.89
*Sexual of Fantasy Questionnaire (SFQ*) [65, 66]	Box A: Fair Box B: NR Box C: NR Box D: NR Box E: Poor Box F: NR Box G: NR Box H: NR Box I: NR	**Construct Validity:** CFA by LISREL in two samples and identified four oblique factors. Factor loadings ≥0.32. In the first sample (*n* = 195): *x*^2^ = 401.64; d.f = 246; GFI = 0.94; NNFI = 0.95; CFI = 0.96; RMSEA = 0.057 In the second sample (*n* = 315): *x*^2^ = 714.76; d.f = 246; GFI = 0.94; NNFI = 0.93; CFI = 0.93; RMSEA = 0.078 **Internal Consistency:** Cronbach’s alpha between 0.66–0.79
*Sexual Desire Conflict Scale for Women (SDCSW)* [18]	Box A: Poor Box B: NR Box C: NR Box D: Poor Box E: Poor Box F: Poor Box G: NA Box H: NR Box I: NR	**Content Validity:** To begin the process of quantitatively assessing the experience of sexual desire and its dysfunctional manifestations, a set of 43 items were written by the first author and also upon clinicians’ reports of working with such patients, most typically the survivors of childhood sexual abuse. **Construct Validity:** EFA and varimax rotation identified three factors. Total variance explained by 46.1%.Eigenvalues of ≥3,24. Factor loadings between 0.30 to 0.84 for the three factors. **Internal Consistency:** Cronbach’s alpha of 0.927.
*Sexual Desire Inventory (SDI-2)* [2]	Box A: Good Box B: NR Box C: NR Box D: Good Box E: Good Box F: Good Box G: NA Box H: NR Box I: NR	**Content Validity:** Items were selected by considering extant theoretical models of desire, diagnostic criteria used in the DSM-III-R for HSDD, and clinical experience in assessing and treating sexual desire disorders. The items were presented initially to sexology researchers and clinicians, who rated the face validity and the clarity of the items. **Construct Validity:** EFA and MLFA Maximum Likehood Factor Analysis. Eigenvalues of ≥1.85 for both factors. With a correlation of *r* = 0.35–0.49, (*p* < 0,001). With total variance explained by 65.11% **Hypothesis testing:** Was hypothesized that the model containing two correlated dimensions would provide a better fit to the data r = 0.35 **Internal Consistency:** Cronbach’s alpha between 0.86–0.96
*Sexual Desire Inventory (SDI-2*) [25]	Box A: Good Box B: Good Box C: NR Box D: Good Box E: Good Box F: NR Box G: Fair Box H: NR Box I: NR	**Construct Validity:** PCA identified two factors. Eigenvalues of 4.66 for the first factor and 1.85 for the second factor. With total variance explained by 64% **Internal Consistency:** Cronbach’s alpha between 0.78–0.87. **Reliability:** Test-Retest: Correlation between r = 0,76–0,83 and r = 0,82 for the global test. **Cross-cultural Validity:** The questionnaire was translated into German by two independent persons (including a native English speaker with excellent German language skills). The two versions were reviewed and revised in several steps for deviations. Thus, taking into account the clarity of the content and the correct German grammar, which was checked by a third person independent of the study, the final German version of the SDI-2 was developed. In terms of content clarity, the synonyms for English-speaking terms used in Germany were taken into account.
*Sexual Desire Inventory (SDI-2*) [26]	Box A: Good Box B: NR Box C: NR Box D: NR Box E: Good Box F: Fair Box G: Poor Box H: NR Box I: NR	**Construct Validity:** CFA and the oblique rotation identified four factors: x_98_^2^ = 299.50, GFI = 0.98, TLI = 0.96, RNI = 0.96, RMSEA = 0.058 attested to two additional factors tested (fantasies and erotophilia). **Hypothesis testing:** Was hypothesized that the model containing two correlated dimensions would provide a better fit to the data *r* = 0.49 (*p* < .001). **Internal Consistency:** Cronbach’s alpha between 0.87–0.88 **Cross-cultural Validity:** Was translated via the parallel-blind technique. This approach requires the participation of at least two people translating an inventory from the source (English) to the target language (Spanish). Then, these translators compare their individual work and collaborate on the final version.
*Menopausal Sexual Interest Questionnaire (MSIQ)* [19]	Box A: Good Box B: Good Box C: NA Box D: Good Box E: Good Box F: Excellent Box G: NA Box H: NR Box I: Good	**Content Validity:** It was designed to focus on sexual desire, responsiveness, and satisfaction in postmenopausal women. The MSIQ was the primary outcome measure of a double-blind efficacy trial. **Construct Validity:** PCA and varimax rotation identified three factors. Total variance explained by 75%. Eigenvalues of 6.44; 1.05; 0.67 respectively. Factor loadings between 0.30 to 0.95 **Hypothesis testing:** For the desire domain of the MSIQ, the correlation coefficient was *r* = 0.82 and for the total score of the MSIQ *r* = 0.81. In contrast, the MSIQ total score was inversely related to the domains in the KSQ and MENQOL: KSQ-Anxiety, − 0.002; KSQ-Depression, − 0.14; and MENQOL-Sexual, *r* = − 0.42. **Reliability:** Test-Retest: Correlation for all items (*r* = 0.52–0.76) and the total correlation scale (*r* = 0.79) **Internal Consistency:** Cronbach’s alpha of 0.87
*The Sexual Interest and Desire Inventory-Female (SIDI-F)* [20, 21, 67, 68]	Box A: Good Box B: Excellent Box C: NR Box D: Excellent Box E: Good Box F: Excellent Box G: NA Box H: NR Box I: NR	**Content Validity:** Was developed as a clinician administered assessment tool to quantify severity of HSDD, and to assess change in response to treatment. A preliminary version of the SIDI-F was presented to 21 volunteers, nine participants without sexual complaint and 12 participants diagnosed with HSDD. This pilot study was carried out in order to assess comprehension and ease of use of the tool, as well as to determine that relevant symptoms were being assessed. A panel of experts in scale development and sexual dysfunction in women from academia and the pharmaceutical industry was convened and met regularly via teleconferences and face-to-face meetings, with e-mail review of ideas and changes [17]. **Construct Validity:** Adequate discrimination in 13 items, however, 5 items were not sensitive to identify the severity of hypoactive sexual desire disorder. Scores ranging from 23 to 58 (average of 37). **Hypothesis testing:** The SIDI-F was highly correlated with the arousal, desire, and satisfaction domains of the FSFI (all correlations *>* 0.8) but not with the lubrication, orgasm, and pain domains. The total score on the SIDI-F was also highly correlated with the arousal, desire/frequency, desire/interest, and pleasure domains of the IVR-CSFQ (all correlations *>* 0.7) but not with the orgasm subscale. [57]. **Internal Consistency:** Cronbach’s alpha of 0,90 **Reliability:** Intraclass Correlation Coefficient (ICC) between 0.85–0.90
*The Sexual Interest and Desire Inventory-Female (SIDI-F)* [28]	Box A: Poor Box B: Fair Box C: NR Box D: Fair Box E: NR Box F: NR Box G: Poor Box H: NR Box I: NR	**Content Validity:** The data collected from face-to-face interviews were checked to determine the participants’ understanding and interpretation of the questionnaire. The qualitative content validity of the Farsi version of SIDI-F was determined by 14 experts in the field of sexual and reproductive health and psychiatry. CVR scores for all items were equal or more than ≥0.79. **Internal Consistency:** Cronbach’s alpha of 0.89 **Reliability:** Intraclass Correlation Coefficient (ICC) between 0.577–0.995 **Cross-cultural Validity:** The inventory was translated into Farsi by two Persian translators. The review and comparison of the two scripts were done by both translators and the head researcher. The final version of the questionnaire was agreed upon after selecting the most appropriate translated phrases, and then two English translators translated the script into English again to ensure the accuracy of the primary translation.
*Cues for Sexual Desire Scale CSDS* [23]	Box A: Good Box B: NR Box C: NR Box D: Good Box E: Excellent Box F: Fair Box G: NA Box H: NR Box I: NR	**Content Validity:** Fifty women (age range 18–67 years) were involved in the item generation stage. The 125 generated items were listed using a conventional questionnaire format. **Construct Validity:** PCA and varimax rotation identified four factors. Factor loadings between 0.43 to 0.89 **Hypothesis testing:** Concurrent validity was assessed by calculating relations between the four factor scores and the total score of the CSDS with the FSFI desire domain scores for women with HSDD (*N* = 30). The different construct revealed *r* = 0.10–0.24 between the FSFI and CSDS. **Internal Consistency:** Cronbach’s alpha between 0.78–0.92
*Cues for Sexual Desire Scale CSDS* [27]	Box A: Good Box B: NR Box C: NR Box D: NR Box E: Excellent Box F: NR Box G: Fair Box H: NR Box I: NR	**Cross-cultural Validity:** The original version of the CSDS was translated into Portuguese by three independent persons fluent in Portuguese and English. The final version was back-translated by a native English speaker. The translation of the English version into Portuguese was semantically equivalent to the English original accordingly to the retro-translation. **Construct Validity:** CFA identified four factors: *x*^2^/d.f. = 24.5; CFI = 0.793; GFI =0.754; RMSEA = 0.08; *P*[RMSEA < 0.05] < 0.001 PCA and varimax rotation identified six factors, but the scree plot extracted five factors. Total explained variance of 58.3% with factorial loads between 0.492–0.854, eigenvalue > 1 and communalities between 0.408–0.750 **Internal Consistency:** Cronbach’s alpha between 0.87–0.90
*Screener for Hypoactive Sexual Desire Disorder in Menopausal Women (SHSDD)* [22]	Box A: Good Box B: Good Box C: NR Box D: Good Box E: Good Box F: NR Box G: NA Box H: NR Box I: NR	**Content Validity:** The tool was then optimized based on feedback from focus groups consisting of physicians and patients in the European Union (Italy, the Netherlands,United Kingdom) and the United States. Each of the four patient focus groups included seven participants over the age of 50 years, who were recruited through general advertising. The four primary care physician focus groups consisted of five participants each. Intrarater reliability with a kappa statistic of 0.97. **Construct Validity:** PCA identified one factor with eigenvalue of 2.98. Total variance explained by 62.9% Factorial loads and communalities were not reported. **Internal Consistency:** Cronbach’s alpha of 0.79 **Reliability:** Intraclass Correlation Coefficient (ICC) of 0.70
*The Sexual Arousal and Desire Inventory (SADI)* [69]	Box A: Good Box B: NR Box C: Good Box D: Fair Box E: Good Box F: Good Box G: NA Box H: NR Box I: NR	**Content Validity:** List of 86 English descriptors was compiled by interviewing approximately 500 men and women. **Construct Validity:** PCA and varimax rotation identified four factors. Total variance explained by 41.3%. Factor loadings ≥0.30 for the total of factors. **Hypothesis testing:** The SADI demonstrated a correlation with an instrument measuring the same construct, MISSA—Sexual Arousal with the SADI—Evaluative *r* = 0.578–0.805 (*p* < 0.01) two tailed. The FS—Positive Affect with the SADI—Motivational and the SADI—Physiological *r* = 0.531–0.554 (p < 0.01) two tailed. The SDI-2 with the SADI—Evaluative and SADI—Motivational *r* = 0.536–0.595 (p < 0.01) two tailed. **Cronbach’s alpha:** 0.905 **Measurement error**: SEM > 0.041
*Female Sexual Desire Questionnaire (FSDQ)* [24]	Box A: Good Box B: NR Box C: NR Box D: Good Box E: Good Box F: Good Box G: NA Box H: NR Box I: NR	**Content Validity:** Preliminary items for the FSDQ were determined through individual interviews with 40 heterosexual partnered women. Interview data were analyzed using the principles of interpretive phenomenological analysis, and questionnaire items were developed to reflect the themes extracted from these data. Questions assessing the DSM-IV-TR criteria for HSDD. Approximately 250 candidate items were peer reviewed by a group of three researchers/clinicians for item appropriateness, relevance, redundancy, and ease of understanding. **Construct Validity:** EFA and the oblimin rotation identified six factors. Total variance explained by 60%. Factor loadings > 0.40 **Hypothesis testing:** The FSDQ demonstrated a correlation with an instrument measuring the same construct, The sexual desire factor the FSDQ with the SDI-2 dyadic domain *r* = 0.78 (*p* < 0.01) two tailed. The solitary desire factor the FSDQ with the SDI-2 solitary domain *r* = 0.83 (p < 0.01) two tailed. **Cronbach’s alpha:** 0.80–0.92

*NA* not applicable, *NR* not reported, Internal consistency (Box A), Reliability (Box B), Measurement error (Box C), Content validity (Box D), Structural validity (Box E), Hypothesis testing (Box F), Cross-cultural validity (Box G), Criterion validity (Box H), Responsiveness (Box I)

**Table 3:** Levels of evidence of the quality of psychometric properties of the instruments of sexual desire

Instrument	Internal Consistency	Reliability	Measurement Error	Validity of Content	Structural Validity	Hypothesis Testing	Cross Cultural Validity	Responsiveness
*Questionnaire Measure of Sexual Interest (QMSI)*	?	?	na	na	?	na	na	na
*Sexual of Fantasy Questionnaire (SFQ)*	++	na	na	na	?	na	–	na
*Sexual Desire Conflict Scale for Women (SDCSW)*	?	na	na	na	?	?	na	na
*Sexual Desire Inventory (SDI-2)*	++	–	na	++	+++	–	–	na
*Menopausal Sexual Interest Questionnaire (MSIQ)*	+	+	na	+	+	+	na	+
*The Sexual Interest and Desire Inventory-Female (SIDI-F)*	+/−	++	na	+/−	+	+	–	na
*Screener for Hypoactive Sexual Desire Disorder in Menopausal Women (SHSDD)*	+	+	na	+	+	na	na	na
*Cues for Sexual Desire Scale CSDS*	+++	+	na	na	+++	–	+	na
*The Sexual Arousal and Desire Inventory (SADI)*	+	na	+	–	+	+	na	na
*Female Sexual Desire Questionnaire (FSDQ)*	+	na	na	+	+	+	na	na

+++ or --- = strong evidence positive/negative result, ++ or -- = moderate evidence positive/negative result, + or - = limited evidence positive/negative result, +/− = conflicting evidence,? = unknown, due to poor methodological quality, na = no information available

## Discussion

### General characteristics of the included instruments

The language of the 10 original identified instruments is predominantly English, with hegemony aimed at women [18–24]. The most tested instruments were the Sexual Desire Inventory SDI-2 [2, 25, 26].

The cultural adaptation process presented limitations related to the insufficient description of this process according to the COSMIN criteria [25–27] Only two studies evaluated the inter-rater and/or intra-rater as an analytical technique for content validity [22, 28].

According to the parameters in the COSMIN checklist, these limitations affected the methodological quality of the identified instruments to measure sexual desire.

### Dimensions and structure

The sample size in psychometric studies is usually performed on the number of items in the instrument. A total of 10 participants per item have been considered sufficient to guarantee the quality of analysis, except for instruments with less than 10 items [29, 30].

There is evidence that 20 or more participants per item can significantly reduce error and inaccuracies in the solution of psychometric models, such as percentage of samples with correct factor structure, average number of items misclassified in the wrong factor, mean error in eigenvalues, mean error in factorial loads, the percentage of analyses that do not converge after 250 interactions, and percentage with Heywood cases [31].

It is likely that instruments with a good fit, but tested with small samples, show instability in measurement and lose their accuracy in other populations and scenarios, especially in studies with less than 300 participants [29].

The limitation in the number of participants imposes that initial minimum parameters of adequacy, such as factorial loads, communalities, and goodness of fit indexes, are higher than in studies with larger samples. This aims at providing increased surety in the quality of the instrument [30, 32] due to an increased imprecision of techniques with small samples.

In 14 of the 31 analysed articles, the relationship between numbers of participants for each instrument item was greater than 20:1. However, no study reported whether the sample size was determined and whether this design also guided the establishment of the model’s minimum parameters. This result corroborates the results of another review in which only 43% of the analyzed articles had information on the size sample of the studied [33].

### Psychometric properties of instruments

Among the instruments assessed, principal component analysis (PCA) was the dominant technique used for construct validity. Of a total of 15 studies, 8 of them analyzed the data using PCA.It is a data reduction method [30, 31], which considers that all items make up the model and, therefore, are not able to explore factors and produce results of the variable latent [34, 35] Thus, the PCA would not represent a real factorial analysis technique [36], in addition to overestimating the variance values explained in 16.4% [31], also generating overestimated factorial loads and communalities [37, 38].

Even in situations where the factors do not correlate and communalities are moderate, the component variance values tend to be high [38]. Other authors [33, 39] complement that studies have systematically shown that PCA is less accurate than factor analysis, especially when the factorial loads are low or close to 0.40 and there are few items per factor/dimension.

The PCA had become common in recent decades, as computers were slow and expensive; it was a fast and cheap method, an alternative to factor analysis [37]. Although the literature has pointed out the limitations and restrictions in the use of PCA, combined with or without the Varimax rotation (orthogonal), the technique has been dominant in validation studies in the last 30 years, accounting for about 60% of these studies [33, 36, 39, 40].

The use of PCA with the Varimax rotation in validation processes has been considered at least a contradictory combination. The PCA considers that all items make up the model even without effectively testing this hypothesis. It assumes, a priori, that the items correlate, because they measure the same latent variable, particularly in psychosocial models. Conversely, the Varimax rotation considers that the items maintain independence between them, and this combination with PCA may increase imprecision in the model. Thus, non-orthogonal rotations (oblique) seem to be adequate in latent psychosocial variables [41].

The studies that conducted the exploratory factor analysis used eigenvalue as the criterion for the definition of factors (dimensions). This analysis configuration corroborates with notes [42] that the PCA’s popularity with the use of eigenvalue above 1 and the Varimax rotation yielded significant results for several classical datasets [43].

Several of the studies showed variance explained as below 60% [29, 30, 32] which indicates the low capacity of the instrument to measure the latent variable. This point is made even more relevant by the predominant use of PCA, which tends to overestimate indicators, and even then, the levels of explained variance were not satisfactory.

None of the studies provided more robust techniques such as the Parallel Analysis [44, 45] considered one of the most accurate and robust techniques for this purpose [33, 36, 39, 46, 47] The justification for its disuse may be in the absence of this technique in most commercial software.

Another fundamental aspect not addressed in the reviewed studies was the testing of data distribution and its normality to the adequacy of the best statistical technique to be used. In contemporary psychometry, this analysis is essential for the quality of the adequacy of psychometric models. All articles used factorial techniques based on the Pearson’s correlation, which is a parametric technique. It should be noted that the distribution of data is rarely normal in psychosocial studies. Thus, the contemporary recommendation is the adoption of the polychoric correlation when normality is violated [48, 49]. The factorial solutions obtained by the presence of polychoric correlation improved accurate reproductions of the measurement model [50, 51]. All studies used the application of Cronbach’s Alpha as a measure of reliability with the obtained values considered acceptable. This coefficient depends on the magnitude of the correlation between items and number of items in the instrument [52].

There is extensive literature criticizing its use without considering the nature and distribution of the data and sample size, mainly in samples with more than 1000 participants [53, 54]. The study by Revelle and Zinbarg (2009) compared 13 reliability indicators and concluded that, in many cases, the Cronbach’s alpha was not indicated. The use of the McDonald’s Omega and Greatest Lower Bound (GLB) is preferable when there is data asymmetry, even in small samples [55]. It is assumed that high Alpha values do not necessarily mean higher reliability and quality of scales or tests, because they can be the result of long scales with parallel and redundant items or generate a restriction in the construct being studied [56]; one should not seek alpha values above 0.90 [52]. Alpha has been usually used more as a measure of internal consistency rather than reliability; it is easy to prove that alpha is not a measure of internal consistency [53]. An even more severe problem is the use of Alpha to remove items because it is not a technique developed for this purpose.

Reliability was evaluated through testing-retesting using the Pearson’s correlation in 11 of the 31 studies analysed. The authors of the 11 studies described the testing-retesting in detail, informing about the sample used, number of measurements, and mean time of instrument use [19, 25, 57]. This procedure is recommended by several psychometrists [30, 41, 52].

However, the use of the Pearson’s correlation for the testing-retesting has been questioned, because it has been deemed inadequate by not considering the systematic differences, and therefore, the systematic error in the measurements [29, 52] Despite this, the predominance of the Pearson’s correlation in the evaluation of testing-retesting was identified without any testing of data normality.

Another relevant point is the use of testing-retesting before construct validation. It is probable that items are discarded with the use of more adequate and robust techniques by not saturating and/or not conforming to the model after the testing-retesting. Thus, one would have attested reliability and would point to a reliable instrument before showing evidence that the instrument actually measures the latent variable that it is proposed to measure. One would attest to the reliability of the instrument that would differ from the final version, especially when the DeVellis 2017 [58] note that the loss of 50% of the items is expected during the validation process of an instrument is taken. Moreover, Bertchold 2016 [59] questions the use of the term reliability in the testing-retesting reinforcing that the Pearson’s correlation is a measure of association and not of reliability.

Another way of clarifying the reliability of an instrument and the possibility of assuring its quality in different contexts is through invariance testing. It was not evidenced in the analyzed studies.

The invariance is an important aspect in the development of a test, especially when using it in heterogeneous populations [60]. The assumptions of invariance answer some points: a) the factorial structure of the instrument is the same in different groups; b) the items that makeup one factor and the instrument have the same importance for different groups; c) scores of one group can be compared to other groups; d) the items present similar measurement errors for different groups; e) the level of variance between factors differ between groups and; f) the covariance between factors is the same between groups [47]. The temporal invariance, which must be investigated with longitudinal delineations is rarely investigated [61].It would be advisable to test other measurement properties for instrument revalidation to assess whether the original instrument construct remains adequately represented over time.

The present review identified different instruments published to measure sexual desire; however, it illustrated several fragilities in the available instruments. According to COSMIN parameters and criteria of evidence, few were submitted to validation procedures with satisfactory results.

Most of the instruments of measurement of sexual desire evaluated in this review were not used in other contexts and by other authors besides in the studies and authors of the original version. Thus, in the validation process of an instrument, it is fundamental to evaluate its reliability outside its original development context. In general, the lack of a description of the process of the cultural adaptation of instruments may hinder their evaluation and selection in future studies.

Regarding the sample size and structure of the analyzed instruments, most of the studies consider a sample based on the ratio of 20:1 and, therefore, reduce imprecision errors in the psychometric models. The testing of the normality distribution of data is fundamental for choosing between parametric or non-parametric analyses techniques. The most tested properties in the analyzed studies are: construct validity analyzed by means of the PCA as the predominant technique; internal consistency evaluated by the Cronbach’s alpha coefficient, and reliability analyzed with the testing-retesting through the Pearson’s correlation.

The availability of validated instruments is paramount, because their application can contribute to the evaluation of sexual health in the population and qualification of the care provided. Conversely, the lack of valid instruments restricts or mitigates the ability to assess sexual desire in individuals, which can result in non-ideal health care.

### Limitations

The databases chosen for conducting this review are comprehensive; however, other databases and gray literature may be incorporated into future reviews.

The results of this review need to be interpreted with caution, because the studies that did not report the methodological quality procedures contemplated in the COSMIN checklist cannot always be assumed as not having it performed by the authors.

## Conclusion

The present systematic review evaluated the methodological quality of the psychometric properties and the level of evidence of instruments that measure sexual desire, published in current databases. A detailed analysis of each study’s procedures and indicators leads us to the following conclusions.

The analysis predominantly showed the lack of detail of methodological procedures, such as limited information on the cultural adaptation process according to the COSMIN criteria and restricted use of analyses techniques for content validation (inter-rater and intra-rater). These problems have extended to cultural adaptation studies.

Limiting aspects in the validation processes of instruments were observed, which have been recurrently reported in the literature. The reasons for the sizing of study participants were rarely identified. Likewise in the validation of the construct, no testing of data normality distribution; reasons for choosing the extraction, retention, and rotation techniques of items; and establishment in the method of the minimum indicators required for the adequacy of the model were described. Reliability was limited to the application of Cronbach’s alpha, even though there were indications of the instability of indexes due to the number of participants, items, and distribution of data normality.

Only one study applied invariance techniques to ensure that the instrument maintained its properties when used with different populations, contexts, and cultures (sex, race, educational level, and religion among others), especially when it is known that sexual desire may suffer strong interference from moral, social, and religious issues.

Considering that some of the selected instruments were developed in the 1970s and that the majority of others are more than 10 years old from the time of development, we observed that none have been followed up with studies revisiting the psychometric properties of the original instrument in order to adapt and update the content of the instrument’s items in the light of contemporary social and cultural changes. This lack of updates can generate biases of prevalence and in answers, because the instrument fails to capture these sociocultural changes.

The limitations found suggest that most of the instruments analysed in these studies require the application of more robust and contemporary techniques as well as improved detailing of the steps and procedures applied, which would ensure their accuracy and stability, and consequently, their application in the academic and/or clinical settings.

## Additional files

Additional file 1: Search strategy (DOCX 14 kb)

Additional file 2: Data extraction form (DOCX 17 kb)

Additional file 3: Supplemental references s1–s21 (DOCX 17 kb)

## Abbreviations

COSMIN: COnsensus-based Standards for the selection of health Measurement INstruments
CSDS: Cues for Sexual Desire Scale
FSDQ: Female Sexual Desire Questionnaire
MSIQ: Menopausal Sexual Interest Questionnaire
PCA: Principal Component Analysis
QMSI: Questionnaire Measure of Sexual Interest
SADI: The Sexual Arousal and Desire Inventory
SDCSW: Sexual Desire Conflict Scale for Women
SDI-2: Sexual Desire Inventory
SFQ: Sexual of Fantasy Questionnaire
SHSDD: Screener for Hypoactive Sexual Desire Disorder in Menopausal Women
SIDI-F: The Sexual Interest and Desire Inventory-Female
