# Supplementary material for: Systematic review of the psychometric properties of instruments to measure sexual desire
Source: BMC Med Res Methodol. 2018 Oct 19;18:109. doi: 10.1186/s12874-018-0570-2 (PMC6194697; doi:10.1186/s12874-018-0570-2)
Supplement: Supplementary file 1 — Search strategy (DOCX 14 kb) [file 12874_2018_570_MOESM1_ESM.docx]

Search strategy

Search: PubMed strategy

1. "Women"[Mesh]
2. "Men"[Mesh]
3. "Woman"
4. "Women"
5. "Men"
6. "Man"
7. or/1-7
8. "Libido"[Mesh]
9. "Sexual Desire"
10. "Sexual Interest"
11. or/ 8-10
12. "Psychometrics"[Mesh])
13. "Validation Studies" [Publication Type]
14. "Validation Studies as Topic"[Mesh]
15. "Reproducibility of Results"[Mesh])
16. "Cross-Cultural Comparison"[Mesh])
17. "Psychometrics"
18. "Validation Studies"
19. "Validation Studies as Topic"
20. "Reproducibility of Results"
21. "Cross-Cultural Comparison"
22. or/12-21

Search: EMBASE strategy

1. 'female'/exp
2. 'male'/exp
3. 'women'
4. 'men'
5. 'woman'
6. 'man'
7. or/1-6
8. 'sexual desire'/exp
9. 'libido'/exp
10. 'sexual interest'/exp
11. 'libido'
12. 'sexual desire'
13. 'sexual interest'
14. or/8 -13
15. 'psychometry'/exp
16. 'reproducibility'/exp
17. 'cross cultural adaptation'/exp
18. 'validation study'/exp
19. 'psychometry'
20. 'reproducibility'
21. 'cross cultural adaptation'
22. 'validation study'
23. 'cross-cultural comparison'
24. 'reproducibility of results'
25. or/15-24

Search: Science Direct strategy

1. "Women"
2. "Men"
3. "Woman"
4. "Man"
5. or/1-4
6. "Libido"
7. "Sexual Desire"
8. "Sexual Interest"
9. or/6-8
10. "Psychometrics"
11. "Validation Studies"
12. "Validation Studies as Topic"
13. "Reproducibility of Results"
14. "Cross-Cultural Comparison"
15. or/10-14

Search: PsycINFO strategy

1. "Woman"
2. "Women"
3. "Men"
4. "Man"
5. or/1-4
6. "Libido"
7. "Sexual Desire"
8. "Sexual Interest"
9. or/6-8
10. {Psychometrics}
11. {Test Validity}
12. "Psychometrics"
13. "Validation Studies"
14. "Validation Studies as Topic"
15. "Reproducibility of Results"
16. "Cross-Cultural Comparison"

Search: Web of Science strategy

1. women*
2. men*
3. or/1-2
4. "Libido"
5. "Sexual Desire"
6. "Sexual Interest"
7. or/4-6
8. Psychometric
