# Supplementary material for: Systematic review of the psychometric properties of instruments to measure sexual desire
Source: BMC Med Res Methodol. 2018 Oct 19;18:109. doi: 10.1186/s12874-018-0570-2 (PMC6194697; doi:10.1186/s12874-018-0570-2)
Supplement: Supplementary file 2 — Data extraction form (DOCX 17 kb) [file 12874_2018_570_MOESM2_ESM.docx]

Data extraction form

| Reviewer |  |
| --- | --- |
| Author |  |
| Year of Publication |  |
| Country |  |
| Title of the study |  |
| Source | ⬜ PubMed  ⬜ Excerpta Medica Database (EMBASE)  ⬜ PsycINFO  ⬜ Science Direct  ⬜ Web of Science |
| Inclusion criteria | Original studies, published in Portuguese, English, and Spanish with human beings, and presenting the process of evaluating cultural validations and adaptations of sexual desire instruments, regardless of sample sex or gender. There was no limitation on the initial date of publication, and studies published until November 2017 were considered. In addition, it was determined that articles presenting the dimension of sexual desire or the condition of its decrease (hypoactive sexual desire disorder) would also be included |
| Exclusion criteria | Articles that aimed to measure dysfunctions in other dimensions of the sexual response in men and/or women and samples with pediatric population were excluded. |
| Items | Number of items of the questionnaire or tool. |
| Average fill time |  |
| Population and Sample size (n) |  |
| Types of psychometric properties tested | ⬜ Internal consistency  ⬜ Reliability  ⬜ Measurement error  ⬜ Content validity  ⬜ Structural validity  ⬜ Hypothesis testing  ⬜ Cross-cultural validity  ⬜ Criterion validity  ⬜ Responsiveness |
