# Supplementary material for: Systematic review of the psychometric properties of instruments to measure sexual desire
Source: BMC Med Res Methodol. 2018 Oct 19;18:109. doi: 10.1186/s12874-018-0570-2 (PMC6194697; doi:10.1186/s12874-018-0570-2)
Supplement: Supplementary file 3 — Supplemental references s1–s21 (DOCX 17 kb) [file 12874_2018_570_MOESM3_ESM.docx]

Supplemental references s1­–s21

1. Carvalheira A, Brotto LA, Maroco J. Portuguese Version of Cues for Sexual Desire Scale: The Influence of Relationship Duration. J Sex Med. 2011;8:123–31. doi:10.1111/j.1743-6109.2010.01909.x.
2. Clayton AH, Goldmeier D, Nappi RE, Wunderlich G, Lewis-D’Agostino DJ, Pyke R. Validation of the Sexual Interest and Desire Inventory-Female in Hypoactive Sexual Desire Disorder. J Sex Med. 2010;7:3918–28.
3. Clayton AH, Segraves RT, Bakish D, Goldmeier D, Tignol J, van Lunsen RHW, et al. Cutoff Score of the Sexual Interest and Desire Inventory-Female for Diagnosis of Hypoactive Sexual Desire Disorder. J Women’s Heal. 2010;19:2191–5.
4. Clayton AH, Segraves RT, Leiblum S, Basson R, Pyke R, Cotton D, et al. Reliability and Validity of the Sexual Interest and Desire Inventory–Female (SIDI-F), a Scale Designed to Measure Severity of Female Hypoactive Sexual Desire Disorder. J Sex Marital Ther. 2006;32:115–35.
5. Goldhammer DL, McCabe MP. Development and Psychometric Properties of the Female Sexual Desire Questionnaire (FSDQ). J Sex Med. 2011;8:2512–21.
6. Harbison JJM, Graham PJ, Quinn JT, McAllister H, Woodward R. A questionnaire measure of Sexual Interest. Arch Sex Behav. 1974;3:357–66.
7. Kaplan HS, Harder DW. The sexual desire conflict scale for women: Construction, internal consistency, and two initial validity tests Psychological Reports. 1991;68:1275–82.
8. Kuhn W, Koenig J, Donoghue A, Hillecke T, Warth M. Psychometrische Eigenschaften einer deutschsprachigen Kurzversion des Sexual Desire Inventory (SDI-2). Zeitschrift für Sex. 2014;27:138–49.
9. Leiblum S, Symonds T, Moore J, Soni P, Steinberg S, Sisson M. Original research—outcomes assessment: A Methodology Study to Develop and Validate a Screener for Hypoactive Sexual Desire Disorder in Postmenopausal Women. J Sex Med. 2006;3:455–64.
10. Malary M, Pourasghar M, Khani S, Moosazadeh M, Hamzehgardeshi Z. Psychometric Properties of the Sexual Interest and Desire Inventory-Female for Diagnosis of Hypoactive Sexual Desire Disorder: The Persian Version. Iran J Psychiatry. 2016;11(4):262–8.
11. McCall K, Meston C. Original research—psychology: Cues Resulting in Desire for Sexual Activity in Women. J Sex Med. 2006;3:838–52.
12. Ortega V, Zubeidat I, Sierra J. Further examination of measurement properties of Spanish version of the sexual desire inventory with Undergraduates and adolescent students. Psychol Rep. 2006;99:147–65.
13. Rosen RC, Lobo RA, Block BA, Yang H-M, Zipfel LM. Menopausal Sexual Interest Questionnaire (MSIQ): A Unidimensional Scale for the Assessment of Sexual Interest in Postmenopausal Women. J Sex Marital Ther. 2004;30:235–50.
14. Sierra J., Martin-Ortiz J., Ortega V. Propiedades psicométricas del cuestionario de Wilson de fantasías sexuales. Rev Mex Psicol. 2004;21(1):37-50.
15. Sierra JC, Ortega V, Zubeidat I. Confirmatory Factor Analysis of a Spanish Version of the Sex Fantasy Questionnaire: Assessing Gender Differences. J Sex Marital Ther. 2006;32:137–59. doi:10.1080/00926230500442318.
16. Sills T, Wunderlich G, Pyke R, Segraves RT, Leiblum S, Clayton A, et al. Original research—women’s sexual dysfunctions: The Sexual Interest and Desire Inventory—Female (SIDI‐F): Item Response Analyses of Data from Women Diagnosed with Hypoactive Sexual Desire Disorder. J Sex Med. 2005;2:801–
17. Spector IP, Carey MP, Steinberg L. The sexual desire inventory: Development, factor structure, and evidence of reliability. J Sex Marital Ther. 1996;22:175–90.
18. Toledano R, Pfaus J. ORIGINAL RESEARCH—OUTCOMES ASSESSMENT: The Sexual Arousal and Desire Inventory (SADI): A Multidimensional Scale to Assess Subjective Sexual Arousal and Desire. J Sex Med. 2006;3:853–77.
19. Wilson GD, Lang RJ. Sex differences in sexual fantasy patterns. Pers Individ Dif. 1981;2:343–6.
20. Wilson GD. Measurement of sex fantasy. Sex Marital Ther. 1988;3:45–55.
21. Wilson GD. The Secrets of Sexual Fantasy. London: J. M. Dent & Sons ltd.; 1978.
